# Estimating the Epidemic Size of Superspreading Coronavirus Outbreaks in Real Time: Quantitative Study

**DOI:** 10.2196/46687

**Published:** 2024-02-12

**Authors:** Kitty Y Lau, Jian Kang, Minah Park, Gabriel Leung, Joseph T Wu, Kathy Leung

**Affiliations:** 1 Laboratory of Data Discovery for Health Limited (D24H) Hong Kong Science Park China (Hong Kong); 2 WHO Collaborating Centre for Infectious Disease Epidemiology and Control, School of Public Health Li Ka Shing Faculty of Medicine The University of Hong Kong China (Hong Kong); 3 Department of Health Convergence Ewha Womans University Seoul Republic of Korea; 4 The University of Hong Kong – Shenzhen Hospital Shenzhen China

**Keywords:** coronavirus, superspreading event, SSE, epidemic size, severe acute respiratory syndrome, SARS, Middle East respiratory syndrome, MERS, coronavirus disease 2019, COVID-19

## Abstract

**Background:**

Novel coronaviruses have emerged and caused major epidemics and pandemics in the past 2 decades, including SARS-CoV-1, MERS-CoV, and SARS-CoV-2, which led to the current COVID-19 pandemic. These coronaviruses are marked by their potential to produce disproportionally large transmission clusters from superspreading events (SSEs). As prompt action is crucial to contain and mitigate SSEs, real-time epidemic size estimation could characterize the transmission heterogeneity and inform timely implementation of control measures.

**Objective:**

This study aimed to estimate the epidemic size of SSEs to inform effective surveillance and rapid mitigation responses.

**Methods:**

We developed a statistical framework based on back-calculation to estimate the epidemic size of ongoing coronavirus SSEs. We first validated the framework in simulated scenarios with the epidemiological characteristics of SARS, MERS, and COVID-19 SSEs. As case studies, we retrospectively applied the framework to the Amoy Gardens SARS outbreak in Hong Kong in 2003, a series of nosocomial MERS outbreaks in South Korea in 2015, and 2 COVID-19 outbreaks originating from restaurants in Hong Kong in 2020.

**Results:**

The accuracy and precision of the estimation of epidemic size of SSEs improved with longer observation time; larger SSE size; and more accurate prior information about the epidemiological characteristics, such as the distribution of the incubation period and the distribution of the onset-to-confirmation delay. By retrospectively applying the framework, we found that the 95% credible interval of the estimates contained the true epidemic size after 37% of cases were reported in the Amoy Garden SARS SSE in Hong Kong, 41% to 62% of cases were observed in the 3 nosocomial MERS SSEs in South Korea, and 76% to 86% of cases were confirmed in the 2 COVID-19 SSEs in Hong Kong.

**Conclusions:**

Our framework can be readily integrated into coronavirus surveillance systems to enhance situation awareness of ongoing SSEs.

## Introduction

In the past 2 decades, 3 coronaviruses have emerged and caused widespread public anxiety: SARS-CoV-1 in 2002; MERS-CoV in 2012; and most recently, SARS-CoV-2 in 2019. As of November 2023, the new COVID-19 caused by SARS-CoV-2 has led to more than 771 million confirmed cases and almost 7 million deaths [1]. Despite the differences in epidemiologic characteristics, all 3 coronaviruses are marked by transmission heterogeneity and the ability to produce disproportionally large clusters via superspreading events (SSEs) [2-10]. For instance, it is estimated that 19% of the cases caused 80% of the local transmission in the first wave of COVID-19 epidemic in Hong Kong by April 28, 2020 [11]. The overdispersed transmission pattern was also previously reported for both SARS and MERS [5,8,12].

SSEs are shaped by a combination of determinants originating from the virus, host, and environment [13]. The emergence of mutations and variants with increased transmissibility and greater immune escape mechanisms contribute substantially to the occurrence of SSEs [14]. Superspreaders, who are known to be unusually infectious, were reported to shed a higher viral load over an extended duration [15], which was exacerbated in the context of COVID-19 because significant proportion of these individuals who are infectious could transmit the virus before symptom onset (presymptomatic) or without showing any symptoms (asymptomatic) [16-20]. Certain environments facilitate the transmission, as evidenced by most SSEs of SARS and MERS being nosocomial outbreaks [2,21].

Although the exact mechanism of SSEs is not well established, monitoring SSEs in real time is critical to contain and mitigate epidemics of coronaviruses [22]. It is estimated that implementing control measures a week earlier would lead to a 2.6 times decrease in average epidemic size and a reduction of 4 weeks in average epidemic duration [21,23]. Real-time estimation of the epidemic size of SSEs allows us to proactively take timely measures, including resource planning and mobilization, testing, contact tracing, and implementing targeted interventions in high-risk settings. However, given the inherent heterogeneity of an epidemic, population-level measurements of the average transmissibility of a typical individual who is infectious, such as basic reproductive number (*R_0_*) or effective reproductive number (*R_t_*), are not appropriate for estimating the transmission potential of SSEs [5]. Here, we developed a framework based on back-calculation for SSE epidemic size estimation, allowing for a more comprehensive understanding of the transmission dynamics.

## Methods

### Ethical Considerations

This study involves secondary analysis of existing aggregate research data, including SARS-CoV-1 data from Leung et al [24], MERS data from Cowling et al [7], and COVID-19 data that are publicly available [25]. All data were deidentified and only aggregate data were used. The University of Hong Kong/Hospital Authority Hong Kong West Institutional Review Board approved the secondary analysis without requiring further consent. As participants were not directly involved in the current research activities, no compensation was provided to the individuals.

### Model Assumptions

Back-calculation was first designed for short-term prediction of diseases with a long incubation period [26] and has also been applied to point-source outbreaks with limited onward transmission, such as Legionnaires disease [27]. We extended the method and focused on estimating the final size of symptomatic or laboratory-confirmed cases at the early stage of a coronavirus SSE, and asymptomatic cases were not considered in the estimation if they were not confirmed and reported.

We assumed that (1) all cases in a given SSE were infected at around the same time (eg, infected by the same superspreader or environmental exposure within a short period of time); (2) all cases followed the same probability distribution function (PDF) of the incubation period, the same PDF of the onset-to-confirmation delay, and the same PDF of the generation time; and (3) there was limited onward secondary transmission when the SSEs were controlled by sufficient contact tracing and timely responses. For SARS and MERS, we assumed that the *R_t_* of secondary transmission was 0, given their relatively lower potential of person-to-person transmission [2]. For COVID-19, which has much higher transmissibility and significant presymptomatic transmission, we assumed that the *R_t_* of secondary transmission could be greater than 1, but the transmission was restricted within 1 disease generation. We assumed that testing and contact tracing policies were the same within 14 days, given that the generation time for SARS-CoV-1, MERS, and ancestral SAR-CoV-2 are around 7 days (Table 1).

**Table 1:** Parameters used in the simulations.

Simulated scenarios	Incubation period (lognormal distribution): mean range (SD range)	Onset-to-confirmation delay (lognormal distribution): mean range (SD range)	Secondary transmission^a^: *R_t_^b^*	Generation time (lognormal distribution): mean range (SD range)	References
SARS and MERS	3-15 (1-10)	—^c^	0	—	[28-32]
SARS and MERS	3-15 (1-10)	0-7 (1-7)	0	—	[33,34]
COVID-19	5-8 (2-5)	—	1.5	5-7 (5-7)	[19,35-38]
COVID-19	5-8 (2-5)	3-7 (2-5)	1.5	5-7 (5-7)	[19]
COVID-19 adjusted for presymptomatic transmission^d^	5-8 (2-5)	—	1.5	5-7 (5-7)	[19,35-38]
COVID-19 adjusted for presymptomatic transmission	5-8 (2-5)	3-7 (2-5)	1.5	5-7 (5-7)	[19]

^a^Secondary transmission can be measured by *R_t_* within 1 disease generation.

^b^*R_t_*: effective reproductive number.

^c^Not applicable.

^d^The 60% presymptomatic transmission was included in the prior information.

**Figure 1:**
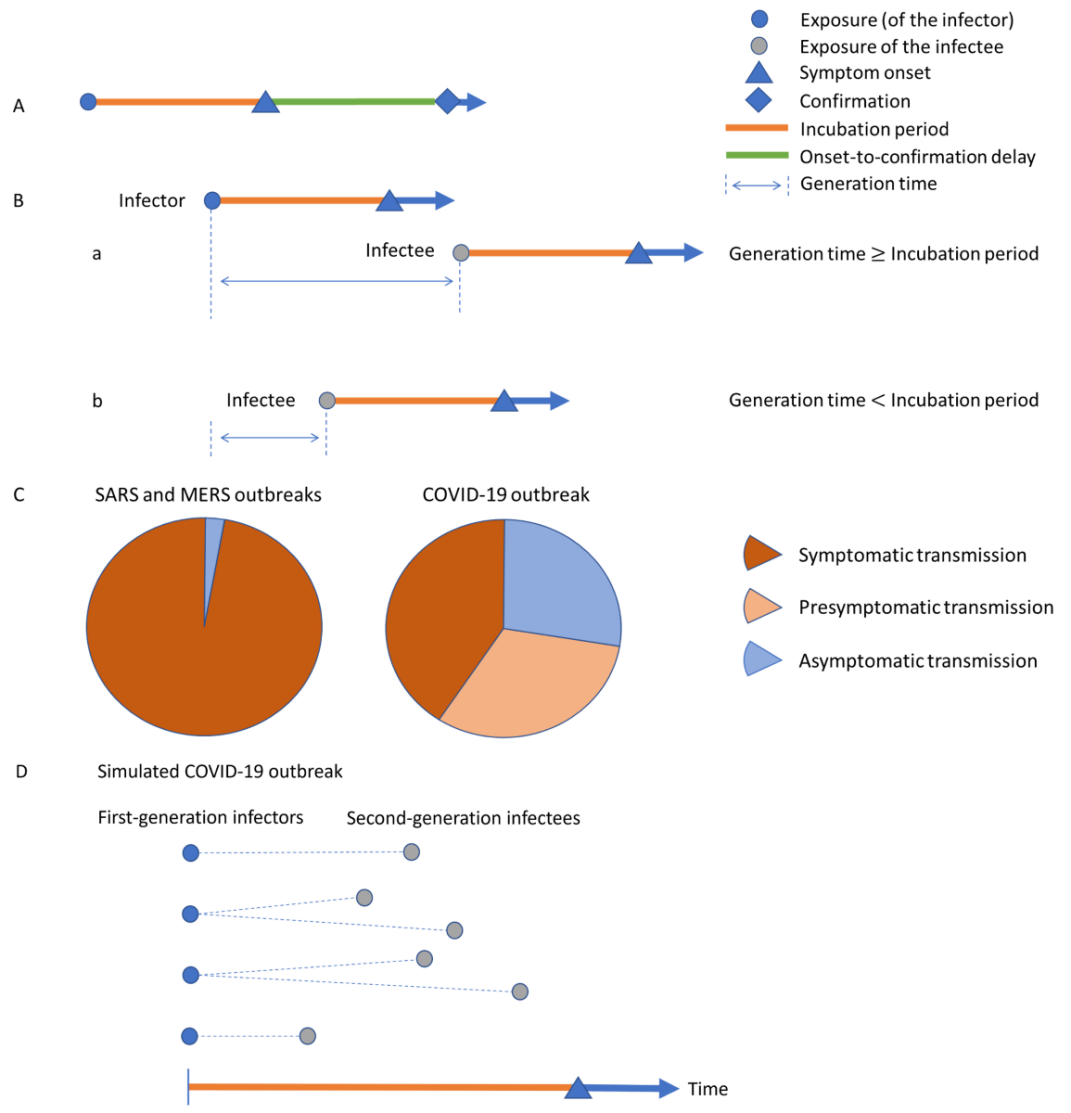
Characteristics of coronavirus SSEs. (A) Time of exposure, symptom onset, and case confirmation. The incubation period is the time interval between infection and symptom onset. The onset-to-confirmation delay is the time interval between symptom onset and confirmation. (B) Transmission between an infector-infectee pair: (a) transmission occurs after symptom onset of the infector (symptomatic transmission); (b) transmission occurs before symptom onset of the infector (presymptomatic transmission). The generation time is the interval between the exposure time of the infector and the infectee. (C) Approximate distribution of presymptomatic, asymptomatic, and symptomatic transmission for SARS, MERS, and COVID-19. (D) Simulated COVID-19 SSE. The undetected presymptomatic transmission pairs within 1 disease generation and *R_t_*=1.5 in a simulated COVID-19 SSE with 10 cases. *R_t_*: effective reproductive number; SSE: superspreading event.

Figure 1A-B summarizes the characteristics of coronavirus SSEs in disease surveillance. Here, the incubation period is the time interval between infection and symptom onset; in practice, there is a delay from symptom onset to case confirmation, and the time of symptom onset can be unavailable for asymptomatic infections or due to underreporting.

### Estimating the Size of SSEs

We formulated the model to estimate the epidemic size of SSEs as follows. Let *X* denote the incubation period with PDF *g*(*x*) and *Y* denote the onset-to-confirmation with PDF *h*(*y*). For an SSE *j*, let *n_j_* be the total number of people infected at time 0 and *k_j_* be the number of symptomatic cases that have been confirmed up to (and including) time *t_j_* since the exposure time for this SSE.

Let *x_i,j_* and *y_i,j_* be the values of *X* and *Y* for the *i*th case in the *j*th SSE. Let *f*(*X,Y*|*θ*) be the joint probability distribution of *X* and *Y* with *θ* representing the parameters. We assume that *X* and *Y* are independent such that *f*(*X,Y*|*θ*) = *g*(*x*)*h*(*y),* and *F*(*X,Y*|*θ*) is the cumulative probability distribution of *f*(*X,Y*|*θ*). The number of symptomatic cases *k_j_* has a binomial distribution with SSE size *n_j_* and success probability *F*(*X*+*Y*>*t*; *θ*). Therefore, the likelihood function for *j*th SSE is:



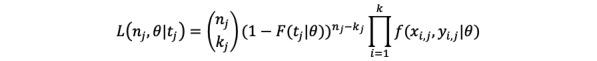



If the time of symptom onset is known, let *M_j,1_* be the set of confirmed cases with known time from exposure to symptom onset and known time from symptom onset to confirmation with a size of *m_j,1_*. If the time of symptom onset is unknown, let *M_j,2_* be the set of confirmed cases with unknown time from exposure to symptom onset but known time from exposure to confirmation with a size of *m_j,2_*. Let *Z* denote *X*+*Y* with the probability distribution *q*(*z*|*θ*). The likelihood function for the *j*th SSE becomes:



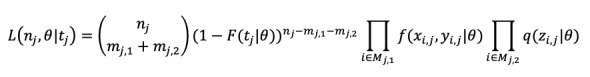



The overall likelihood function for *w* SSEs is:



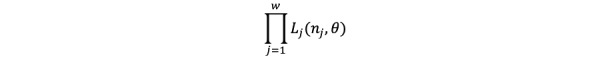



We estimated the SSE size {*n_j_*} and other parameters *θ* using Markov chain Monte Carlo with Gibbs sampling and noninformative flat priors. We considered an estimate to be accurate if the 95% credible interval (Crl) covered the true value. An estimate was precise if the Crl had a relative error within 1.

### Model Validation

We simulated SSE scenarios based on the epidemiological characteristics of SARS, MERS, and COVID-19 (Figure 1C). For SARS and MERS to be considered under control, we assumed that there was a single exposure and no secondary transmission. For COVID-19, we assumed that 60% of the transmission occurred during the presymptomatic phase [19,20,35,39,40]. For example, if we simulated a single-exposure COVID-19 SSE with 10 cases, 6 (60%) second-generation cases were infected by the other 4 first-generation cases in the SSE during the presymptomatic phase. However, the 6 second-generation cases would be taken as first-generation infections directly linked to the primary exposure when the presymptomatic transmission was undetected. With limited onward secondary transmission within 1 disease generation, *R_t_* = 6/4 = 1.5 (Figure 1D). Details about the estimation of COVID-19 SSE sizes and the adjustment for presymptomatic transmission are described in Multimedia Appendix 1.

We used the Latin-hypercube sampling to generate 6 simulated coronavirus scenarios. The parameters for the incubation period, onset-to-confirmation delay, and generation time were sampled from the ranges observed from major SSEs of SARS, MERS and COVID-19 (Table 1). Since most of these 3 coronavirus SSEs were reported to consist of 10 to 300 cases [8,11], we first simulated 1000 stochastic SSEs of 30, 50, 100, and 200 infections with and without onset-to-confirmation delay (ie, 8000 SSE scenarios). We assumed that prior distributions of the incubation period and onset-to-confirmation delay could be estimated by bootstrapping from 50 or 100 cases before the occurrence of the simulated SSEs.

The SSE size estimation was performed starting from the time when 5% of all cases were observed until the time when 95% of all cases were observed. To compare scenarios with different epidemic sizes, we used relative size (ie, percentage of the cases observed in an SSE) and SSE duration (ie, percentage of the duration between the time of confirmation of the first and last case) in the assessment of the accuracy of the estimation.

### Case Studies

We applied our framework to the Amoy Gardens SSE for SARS in Hong Kong, nosocomial SSEs for MERS in South Korea, and 2 SSEs from the COVID-19 pandemic in Hong Kong. An alert would be issued when 10 or more cases were confirmed to link with the same index case, by which we assumed that we had minimal data to start the SSE size estimation.

#### Hong Kong Amoy Gardens SARS SSE

The Amoy Gardens SSE was the largest cluster in the 2003 Hong Kong SARS outbreak [4,24]. The index case developed symptoms on March 13-14, was admitted to the Prince Wales Hospital on March 15, and was discharged on March 19. He visited and stayed 1 night at the Amoy Gardens apartment complex, where he had diarrhea. Thus, we assumed that all cases in the Amoy Gardens SSE were exposed to SARS-CoV-1 on March 19. We used noninformative prior distribution for all parameters in the inference since no information about the incubation period and the onset-to-confirmation delay could be estimated before the Amoy Gardens SSE (Table 2). A total of 311 laboratory-confirmed cases were retrospectively confirmed and reported in the Amoy Gardens SSE.

**Table 2:** Prior distributions for parameters.

Case studies	Prior distribution for the incubation period (lognormal distribution): mean (SD) or mean range (SD range)	Prior distribution for the onset-to-confirmation delay (lognormal distribution): mean (SD) or mean range (SD range)	References
SARS Amoy Gardens SSE^a^ in Hong Kong	3-15 (1-10)	0-7 (1-7)	[28-34]
MERS nosocomial SSEs in South Korea	6.3 (4.3)	5.6 (5.4)	[41-44]
COVID-19 restaurant SSEs in Hong Kong	5.2 (3.9)	5.3 (4.0)	[45]

^a^SSE: superspreading event.

#### South Korea Nosocomial MERS SSEs

The MERS SSEs in South Korea in 2015 were the largest MERS outbreak outside of the Middle East, with 82.3% of all confirmed infections caused by only 5 (2.7%) cases [41,46]. The index case visited multiple clinics after traveling to the Middle East. He first developed fever and myalgia illness on May 11 and visited the same clinic on May 12, 14, and 15. The symptoms were not resolved and a cough developed on May 15. The index case was admitted to a secondary hospital (Cluster 1) on May 15 and transferred to a tertiary hospital in Seoul (Cluster 2) on May 17, where he was later diagnosed with MERS-CoV on May 20. Meanwhile, another large SSE took place in other hospitals and was traced back to this same index case (Cluster 3). We took May 15, the day that the index case visited Cluster 1, as the time of exposure of all cases in Cluster 1. In the series of nosocomial SSEs, the time of exposure would be different for each confirmed case depending on when they visited the contaminated hospitals. Therefore, 3 major nosocomial clusters (Cluster 1-3) with sizes of 29, 125, and 27 were studied retrospectively [7,41]. We used MERS data from the previous Middle East outbreaks as the prior information for parameter estimation (Table 2).

#### Hong Kong COVID-19 SSEs

For the COVID-19 case study, 2 linked Hong Kong restaurant SSEs in 2020 with relatively exclusive contact tracing were selected [25]. The first SSE took place at a restaurant of Tao Heung Holdings on July 9 where people gathered in a celebration event, resulting in an SSE of 42 cases. Soon, another SSE took place at another restaurant of Fulum Holdings on July 11, where hundreds of people attended a birthday party, resulting in an SSE of 44 cases. We assumed the time of exposure to be July 9 and July 11 for the 2 SSEs, respectively. Prior information on the incubation period and onset-to-confirmation delay from previous COVID-19 confirmed cases were used in the parameter estimation (Table 2).

## Results

### Model Validation

**Figure 2:**
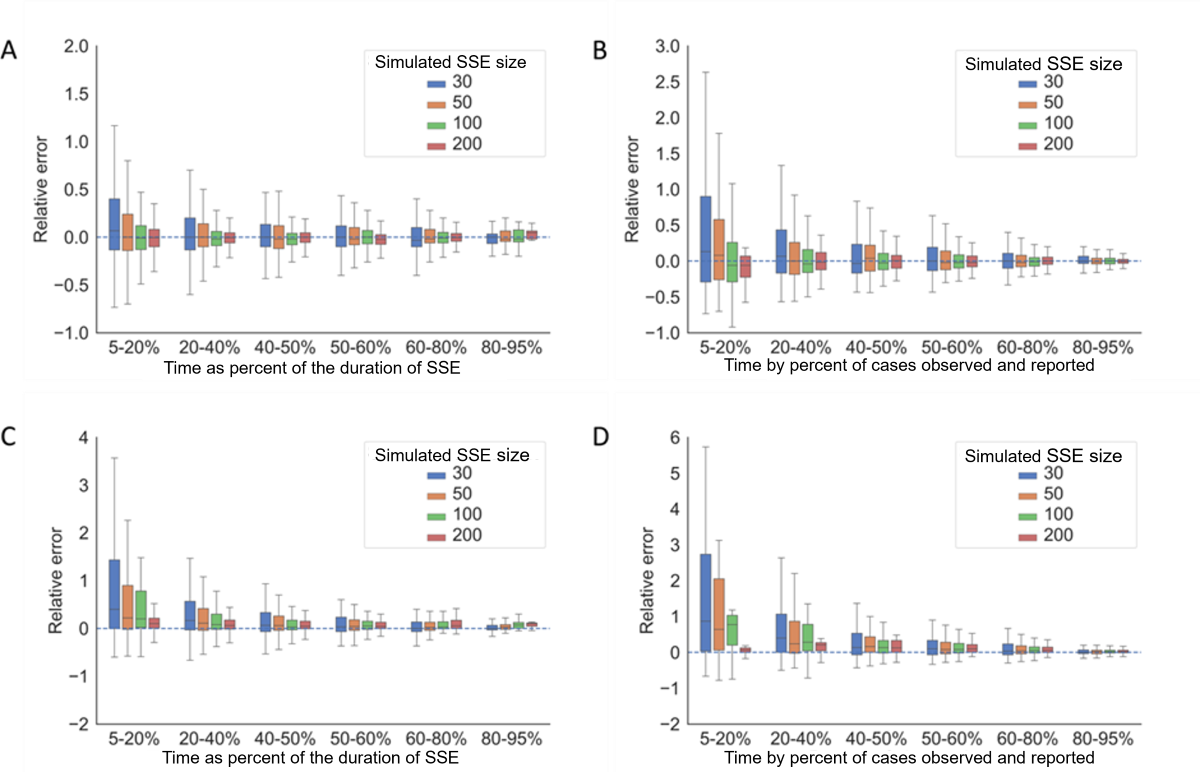
The accuracy and precision of SSE size estimation for simulated SARS and MERS scenarios. Scenario 1: (A) simulated SARS and MERS SSEs with no onset-to-confirmation delay, with size estimation performed at the time as the percentage of the duration of the SSE; and (B) simulated SARS and MERS SSEs with no onset-to-confirmation delay, with size estimation performed at the time by the percentage of cases observed and reported. Scenario 2: (C) simulated SARS and MERS SSEs with onset-to-confirmation delay, with size estimation performed at the time as the percentage of the duration of the SSE; and (D) simulated SARS and MERS SSEs with onset-to-confirmation delay, with size estimation performed at the time by the percentage of cases observed and reported. SSE: superspreading event.

**Figure 3:**
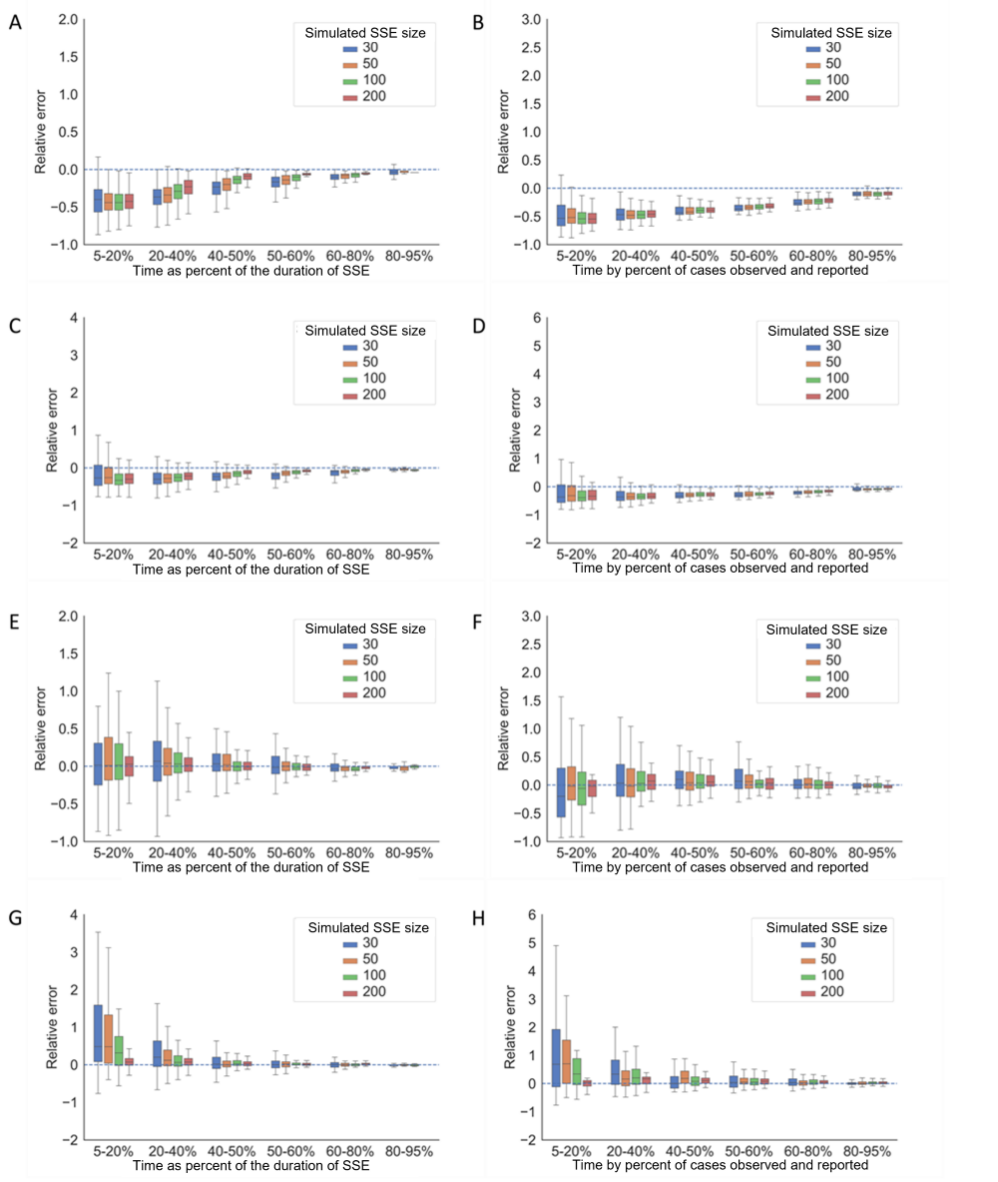
The accuracy and precision of SSE size estimation for simulated COVID-19 scenarios. Scenario 3: (A) simulated COVID-19 SSEs with no onset-to-confirmation delay but 60% presymptomatic transmission, with size estimation performed at the time as the percentage of the duration of the SSE; and (B) simulated COVID-19 SSEs with no onset-to-confirmation delay but 60% presymptomatic transmission, with size estimation performed at the time by the percentage of cases observed and reported. Scenario 4: (C) simulated COVID-19 SSEs with both onset-to-confirmation delay and 60% presymptomatic transmission, with size estimation performed at the time as the percentage of the duration of the SSE; and (D) simulated COVID-19 SSEs with both onset-to-confirmation delay and 60% presymptomatic transmission, with size estimation performed at the time by the percentage of cases observed and reported. Scenario 5: (E) simulated COVID-19 SSEs with no onset-to-confirmation delay but 60% presymptomatic transmission adjusted in prior information, with size estimation performed at the time as the percentage of the duration of the SSE; and (F) simulated COVID-19 SSEs with no onset-to-confirmation delay but 60% presymptomatic transmission adjusted in prior information, with size estimation performed at the time by the percentage of cases observed and reported. Scenario 6: (G) simulated COVID-19 SSEs with onset-to-confirmation delay and 60% presymptomatic transmission adjusted in prior information, with size estimation performed at the time as the percentage of the duration of the SSE; and (H) simulated COVID-19 SSEs with onset-to-confirmation delay and 60% presymptomatic transmission adjusted in prior information, with size estimation performed at the time by the percentage of cases observed and reported. SSE: superspreading event.

Inferred from the model simulation, we found that the accuracy and precision of our estimation increased with the size of the SSE (Figures 2 and 3). For SARS and MERS, with and without onset-to-confirmation delay, all Crls covered the true SSE size and the relative error was less than 1 for all simulated scenarios, when the estimation was performed at the time exceeding 50% of the duration of the SSE or when more than 60% of the cases were observed (Figure 2A**-**B). With the presence of onset-to-confirmation delay, the range of relative error doubled when the estimation was performed before 50% of the duration of the SSE or when less than 60% of the cases were observed (Figure 2C**-**D). Our method tended to overestimate the size in the early stage of an SSE when less than 20% of cases were reported, especially in the scenarios with onset-to-confirmation delay.

If the presymptomatic transmission was not adjusted in the COVID-19 simulated SSEs, there would always be an underestimation even after 50% of the duration of the SSE or when more than 60% of the cases were observed (Figure 3A-D). For a typical COVID-19 SSE with 50 cases, the model would predict the final size to be around 30 when half of the cases were observed if second-generation infections cannot be distinguished from first-generation infections. If we could detect the transmission pattern of COVID-19 in time and adjust for presymptomatic transmission as per our prior information, the estimation would perform similarly well to the simulated scenarios of SARS and MERS when presymptomatic transmission was not included (Figure 3E-H).

### Case Studies

**Figure 4:**
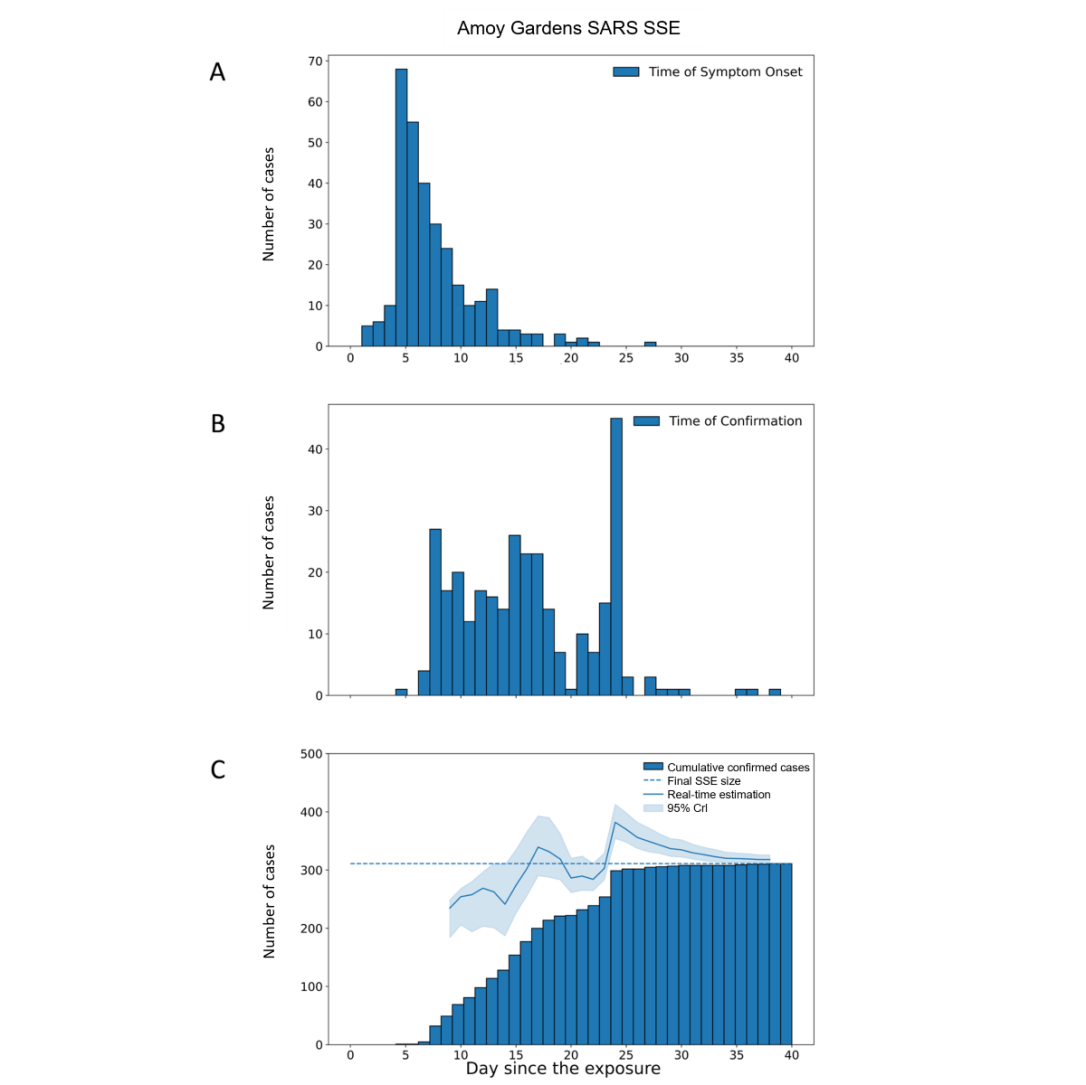
Real-time size estimation of Amoy Gardens SARS SSE in Hong Kong in 2003. (A) Epidemic curve by time of symptom onset. (B) Epidemic curve by time of confirmation. (C) Real-time estimation of SSE size by the time since the exposure (May 19, 2003). The dash line indicates the actual epidemic size of the SSE. Crl: credible interval; SSE: superspreading event.

In the case study of Amoy Gardens SARS SSE, the Crl covered the final SSE size and had a relative error within 1 in 4 days after the alert was issued, which was 25 days before the last case of the SSE was observed (Figure 4). At the time when the alert was issued, only 36.7% (114/311) of all cases were observed. The SSE size estimation became more accurate and precise thereafter.

**Figure 5:**
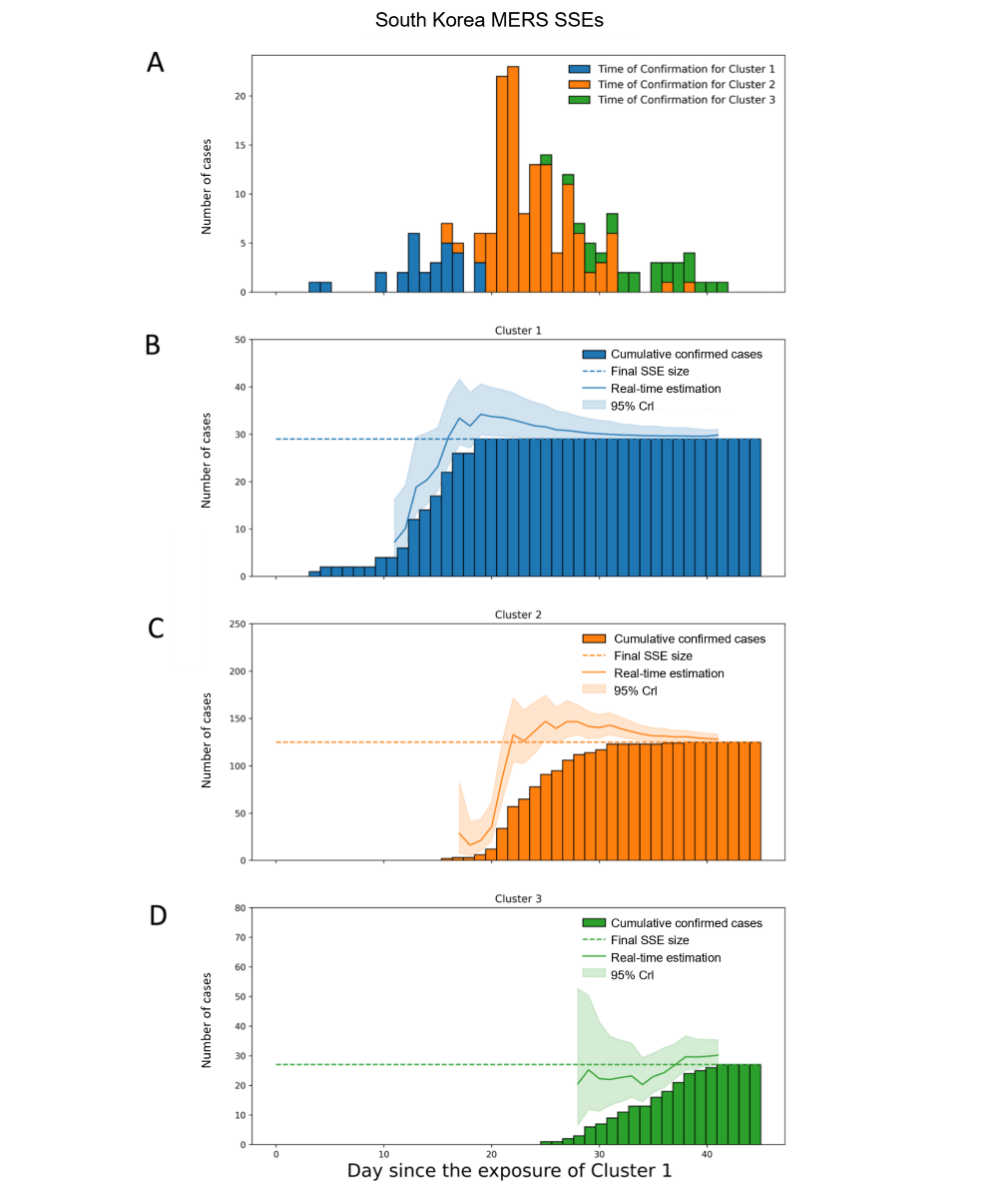
Real-time size estimation of nosocomial MERS SSEs in South Korea in 2015. (A) Epidemic curve by time of confirmation for Cluster 1, Cluster 2, and Cluster 3 denoted by blue, red, and yellow, respectively. (B) Real-time estimation of the SSE size of Cluster 1. (C) Real-time estimation of the SSE size of Cluster 2. (D) Real-time estimation of the SSE size of Cluster 3 by the time since the index case visited Cluster 1 (May 15, 2015). The dash line indicates the actual epidemic size of the SSEs. Crl: credible interval; SSE: superspreading event.

For the series of South Korea MERS SSEs, the Crls of Cluster 1, Cluster 2, and Cluster 3 covered the final SSE sizes with a relative error within 1 in 2 days, 3 days, and 0 days after the alert was issued, respectively, which were 4 days, 15 days, and 9 days before the last case of the respective SSE was observed (Figure 5). At the time when alerts were issued, 62% (18/29), 52% (65/125), and 41% (11/27) of all cases in the respective SSEs were observed, and the estimation converged sooner for later SSEs.

**Figure 6:**
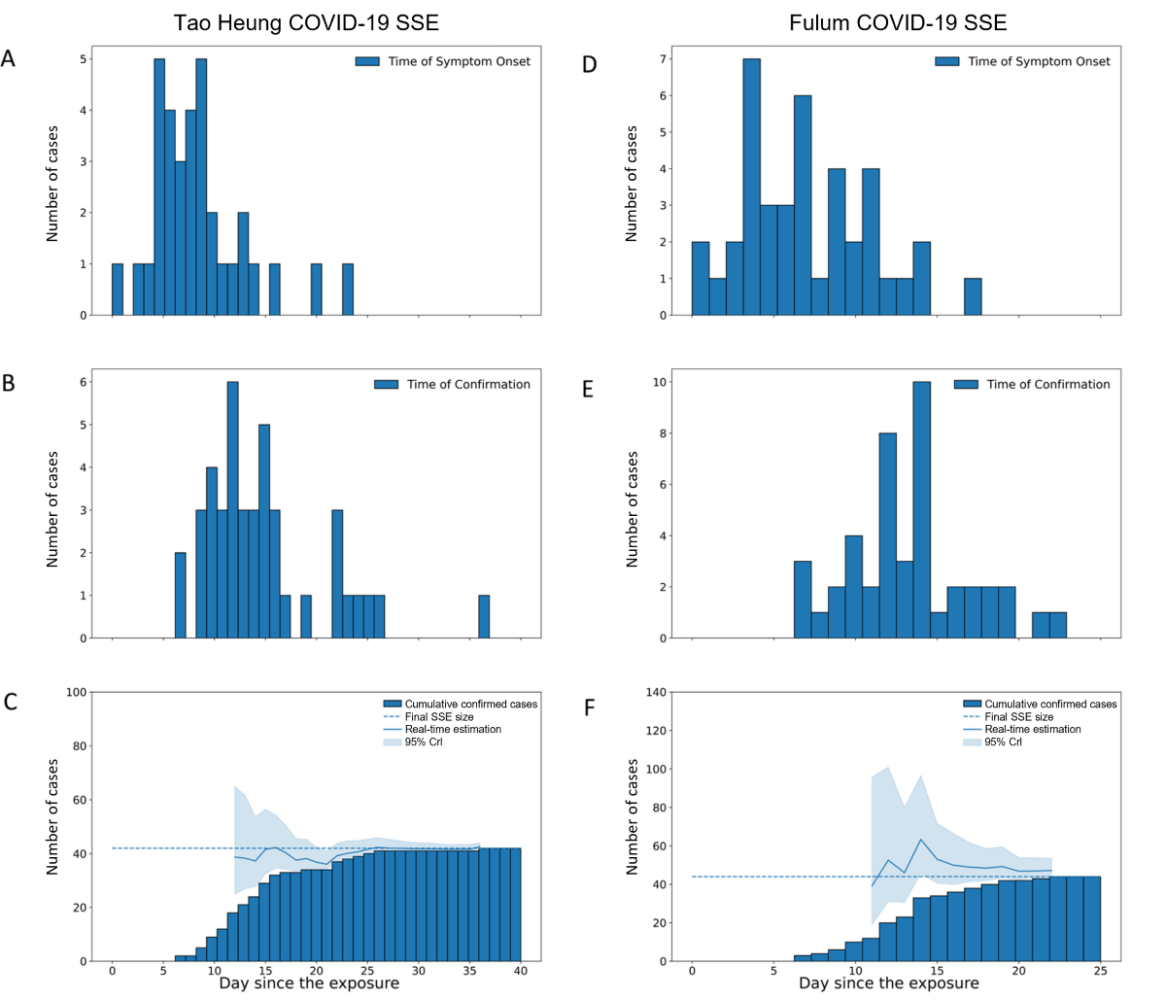
Real-time size estimation of COVID-19 SSEs in 2 restaurants in Hong Kong in 2020. (A) Epidemic curve by time of symptom onset of the Tao Heung SSE. (B) Epidemic curve by time of confirmation of the Tao Heung SSE. (C) Real-time estimation of the size of the Tao Heung SSE by the time since the social gathering on July 9, 2020. (D) Epidemic curve by time of symptom onset of the Fulum SSE. (E) Epidemic curve by time of confirmation of the Fulum SSE. (F) Real-time estimation of the size of the Fulum SSE by the time since the social gathering on July 11, 2020. The dashed line indicates the actual epidemic size of the SSEs. Crl: credible interval; SSE: superspreading event.

For the Tao Heung COVID-19 SSE in Hong Kong, the Crl covered the final SSE size and had a relative error within 1 in 5 days after the alert was issued, which was 20 days before the last case of the SSE was observed. At this time, 76% (32/42) of all cases were observed (Figure 6). Similarly, for the Fulum COVID-19 SSE, the Crl covered the final SSE size and had a relative error within 1 in 7 days after the alert was issued and 5 days before the last case was observed. At the time of issuing the alert, 86% (38/44) of all cases had been observed (Figure 6).

## Discussion

In this study, we developed a framework based on back-calculation to estimate the epidemic size of coronavirus SSEs in real time. Our method bypassed the complexity of developing a transmission model and gave accurate estimates when there were limited secondary transmission within the SSEs. As expected, we found that the precision and accuracy of the size estimation increased with more observed cases, where large SSEs could be spotted earlier, such that immediate responses could be taken. The estimation performed better with the absence of onset-to-confirmation delay, and prior information on parameters improved the performance of the model. In the case study of MERS nosocomial SSEs, we used data from previous MERS cases from the Middle East to inform the estimation in South Korea. Accurate estimation was obtained sooner for Cluster 2 and Cluster 3 with more information about the distribution of the incubation period and the distribution of the onset-to-confirmation delay obtained after the occurrence of Cluster 1.

We showed that the epidemic size of coronavirus SSEs could be accurately estimated before 50% of the cases were reported when there was no undetected secondary transmission, such as in the SARS and MERS simulated scenarios, or when the secondary transmission was adjusted, such as in the COVID-19 simulated scenarios. In the retrospective study on the Amoy Gardens SARS SSE, we got a robust estimation as early as having only 37% of all the cases identified, and in practice, hospitals could prepare resources beforehand for such a large SSE. The estimation of SARS and MERS SSE sizes were more accurate compared with that of COVID-19 because most symptomatic infections would be confirmed and reported. For example, in the case study of Amoy Gardens, it was suspected that SAR-CoV-1 was excreted in the stool and transmitted through sewerage, and the SSE was identified early when many Amoy Gardens cases were traced back to the block where the index case had stayed [4,24].

However, our method tended to overestimate the size early in simulated SARS and MERS SSEs when the observed case number was low. For simulated COVID-19 SSEs, our method always underestimated the size when secondary transmission within the SSE was not detected or observed until later in the SSE. The real-time daily estimation could also be unstable, especially when little was known about the distribution of the incubation period and reporting delays. For example, in the Amoy Gardens SARS SSE, the time of exposure of some cases could be later than the common exposure time that we assumed (May 19, 2003), depending on the time they came into contact with the contaminated areas. The SARS-CoV-1 virus could also be transmitted via person-to-person contact or contamination of communal facilities such as elevators and doors other than sewage [47]. In the MERS case study, we bracketed the window of the exposure time and grouped the cases into 3 hospital SSEs based on the time of visiting specific hospitals.

The recall bias can be another source of errors, especially for COVID-19 infections where symptoms were relatively mild for many cases. Although we explored the source of uncertainty in our estimation, the required accuracy and precision depends on the potential impact of the SSEs, the available resources for control measures, and the requirement for decision-making. For example, to plan resource allocation and better prepare the health care system, the 95% upper bound of the size estimate should be used. For surveillance purposes, the 95% lower bound of the estimate can be used as a threshold to issue alerts and initiate actions. It could also be used as a conservative estimate of the minimal number of infections.

In summary, our framework can be applied to coronavirus SSEs when there is limited undetected secondary transmission or when secondary infection is accounted for. The emergence of more transmissible variants further complicates the situation [48], and intensive contact tracing and testing might be required to alert SSEs in time. Currently, with the COVID-19 pandemic transiting to the endemic phase and society returning to normalcy, our method can be integrated into coronavirus surveillance systems to monitor potential SSEs in large social gatherings.

## Appendix

Multimedia Appendix 1 Details about the estimation of COVID-19 superspreading events and the adjustment for presymptomatic transmission.

## Abbreviations

CrI: credible interval
PDF: probability distribution function
R_0_: basic reproductive number
R_t_: effective reproductive number
SSE: superspreading event

## Data Availability

All the epidemiological information we used is available in the main text or Multimedia Appendix 1. The data, codes, and parameters used to plot Figures 2, 3, 5, and 6 are shared on a GitHub repository [49]. The data (the time of exposure, the time of symptom onsets, the time of case confirmation, the hospital cluster, the calculated incubation period, and the calculated onset-to-confirmation delay) of MERS and COVID-19 case studies were from the Korea Centers for Disease Control and Prevention (KCDC), World Health Organization (WHO), and Centre for Health Protection (CHP) in Hong Kong, which are publicly available. Requests for data of the SARS case study should be sent to the corresponding author.
