# Supplementary material for: Estimating the Epidemic Size of Superspreading Coronavirus Outbreaks in Real Time: Quantitative Study
Source: JMIR Public Health Surveill. 2024 Feb 12;10:e46687. doi: 10.2196/46687 (PMC10863650; doi:10.2196/46687)
Supplement: Multimedia Appendix 1 [file publichealth_v10i1e46687_app1.docx]

**Multimedia Appendix 1**

**Application of the model to coronavirus SSEs with secondary infections**

For COVID-19 simulated scenarios, secondary transmission occurred at the pre-symptomatic phase of first-generation infectors was not detected and taken as having the same point-source exposure as the first-generation symptomatic transmission. We formulated the model as follows.

The observed incubation period $X’$for second-generation infectees due to pre-symptomatic transmission is the interval between the time of exposure of the first-generation infectors and the time of symptom onset of second-generation infectees, which equals to the sum of the actual incubation period $X$ of the second-generation infectees and the generation time $S$. The probability distribution function (PDF) of the observed incubation period for second-generation infectees is denoted as $g’(x’)=g’(x+s)$.

In the estimation, first- and second-generation infections cannot be distinguished. We assumed that they share the same distribution of incubation period and the same distribution of onset-to-confirmation delay, i.e., the joint PDF of $f’(X’, Y’|\theta)$. $F’(X’, Y’|\theta)$ is the cumulative PDF of $f’(X’, Y’|\theta)$.

If the time of symptom onset is known, let $M_{j,1}$be the set of confirmed cases of the first-generation infectors with known time from exposure to symptom onset and known time from symptom onset to confirmation with a size of $m_{j,1},$and let $M_{j,3}$ be the set of confirmed cases of the second-generation infectees resulting from pre-symptomatic transmission of first-generation infectors, with known time from exposure to symptom onset and known time from symptom onset to confirmation with a size of $m_{j,3}$.

If the time of symptom onset is unknown, let $M_{j,2}$ be the set of confirmed cases of the first-generation infectors with unknown time from exposure to symptom onset but known time from exposure to confirmation with a size of $m_{j,2},$ and let $M_{j,4}$ be the set of confirmed cases of the second-generation infectees resulting from pre-symptomatic transmission of first-generation infectors, with unknown time from exposure to symptom onset and known time from exposure to confirmation with a size of $m_{j,4}$. Let $Z’$denotes $X’+ Y’$ with PDF $q’(z’|\theta).$

The number of confirmed cases has a binomial distribution with success probability $F’(X’+Y’>t;\theta)$. In $j$th SSE, the likelihood function becomes

$$L'\left( n_{j},\theta|t_{j} \right)=\binom{n_{j}}{m_{j,1}+m_{j,2}+m_{j,3}+m_{j,4}}\left( 1-F'\left( t_{j} | \theta\right) \right)^{n_{j}-m_{j,1}-m_{j,2}-m_{j,3}-m_{j,4}}$$

$$\prod_{i\in\left\{ M_{j,1},M_{j,3} \right\}} f'({x'}_{i,j},{y'}_{i,j}|\theta)\prod_{i\in\left\{ M_{j,2},M_{j,4} \right\}} q'({z'}_{i,j}|\theta)$$

**Adjusting the priors for coronavirus SSEs with secondary infections**

We assumed that we had the prior information that there was 60% pre-symptomatic transmission in the COVID-19 simulated SSEs. However, the pre-symptomatic transmission remained unobserved and unreported. To adjust the priors, we bootstrapped the distribution of the prior incubation period from 50 simulated cases: 20 (40%) first-generation infectors that have the actual prior incubation period, and 30 (60%) second-generation infectees resulted from pre-symptomatic transmission that have an observed prior incubation period, which equals to the sum of the actual prior incubation period and the generation time.
